# Evaluating Critical Appraisal Skills by Introducing Journal Clubs to Preclinical Dental Students Using the Assessing Competency in Evidence-Based Medicine (ACE) Tool Through Pre and Post-testing

**DOI:** 10.7759/cureus.31535

**Published:** 2022-11-15

**Authors:** Sadaf Mumtaz, Sohail Sabir

**Affiliations:** 1 Medical Education and Simulation, Dental College, HITEC Institute of Medical Sciences, National University of Medical Sciences, Rawalpindi, PAK; 2 Transplant Nephrology, Armed Forces Institute of Urology/Army Medical College, National University of Medical Sciences, Rawalpindi, PAK

**Keywords:** communication skills, decision making, active learning, critical appraisal, journal clubs

## Abstract

Background

The ineffective utilization of journal clubs (JCs) for pre-clinical dental students has led to a lack of research into their effectiveness in developing skills such as critical reasoning and evidence-based medicine (EBM) practice. Therefore, we have implemented JCs in first-year undergraduate dental students and measured their effectiveness using the integrated Assessing Competency in Evidence-Based Medicine (ACE) tool.

Methodology

We conducted a quasi-experimental study where EBM was included in the curriculum for pre-clinical students as a hybrid model with a year-long blended learning approach. The 50-student class was divided into five groups of 10 students, with each group participating in seven JCs related to the physiology curriculum. After conducting critical analysis in self-directed learning sessions, students created interactive PowerPoint presentations followed by discussion. Instructors offered feedback after each session based on 1-2 levels in Kirkpatrick’s training evaluation model. Inferential statistics were used for comparative analysis of the ACE tool pre- and post-test using SPSS version 26 (IBM Corp., Armonk, NY, USA).

Results

A linear trend in median score from 6 in the pre-test to 9 in the post-test was detected using the box and whisker plot. Using paired sample t-test, the mean difference (95% confidence interval) between the pre-test and post-test responses was -3.14 (-2.32 to -3.96) (p < 0.001). In terms of the post-test responses, each item’s difficulty index ranged from 0.3 to 0.9. Internal reliability was in the acceptable range of >0.15 (range = 0.5-0.18). The item discriminatory index was in the range of 0.8 to >0.2. Cronbach’s alpha was 0.64, which was deemed acceptable.

Conclusions

Our results show that pre-clinical dentistry students appreciated the use of JCs to improve active learning, critical appraisal, analytical, and decision-making skills. The 15-item ACE measure is a useful and reliable tool for assessing dentistry students’ EBM proficiency in Pakistan.

## Introduction

Evidence-based medicine (EBM) is critical for the advancement of healthcare professionals in today’s complex healthcare system [1]. EBM follows a five-step approach that involves (i) posing a question that can be answered, (ii) searching the literature, (iii) critical assessment, (iv) applying the evidence, and (v) performance evaluation [1,2]. EBM is incomplete without critical appraisal. It is a self-directed process that is frequently hampered by a lack of instruction and a lack of research time during the pre-clinical and clinical years [3].

The appraisal and critique of evidence is a critical thinking skill described by Bloom’s taxonomy [4]. The use of questioning, class discussions, debates, and written assignment tools for critical thinking is reported to be limited in the literature [5-7]. Many institutions throughout the world are now using problem-based learning to teach critical thinking abilities [7]. Many people believe that journal clubs (JCs) are an important element of postgraduate (PG) health education because they foster the development of critical assessment abilities [5]. They help both dental and medical practitioners evaluate new studies more effectively. However, there is little evidence that JCs play a role in undergraduate (UG) dental education [6,8,9].

The use of JCs for teaching EBM, including research methodology, statistical analysis, and critical appraisal, is strongly recommended by the literature [10-12]. A thorough and methodical evaluation of the reliability or rigor of a study is known as critical appraisal [13]. A study based on 18 studies (17 observational and one randomized controlled trial) the BEME guidance number 16 in 2011 [14] examined the use of JCs in the instruction of EBM. The study concluded that JCs created to teach EBM to healthcare professionals improved readers’ reading habits, critical appraisal, and practical application of findings [14].

According to published studies, EBM, including critical appraisal, is mostly taught during the clinical years of medical school and is frequently hampered by a lack of training and limited research time [6,9]. Retrieving and using evidence was included as an essential ability for graduating medical students in the Association of American Medical Colleges’ Core Entrustable Professional Activities (EPAs) for Entering Residency. Defining a “uniform core set of behaviors that could/should be anticipated of all medical school graduates” is the goal of the program known as Core EPAs for Entering Residency [15]. Medical/Dental students must complete certain educational interventions that focus on developing their critical appraisal skills to become proficient EBM practitioners [6]. According to a few studies conducted in the West, teaching EBM to first-year medical students involved employing hypothesis-based thinking [16], including an eight-hour EBM course [17] and a pilot EBM program focused on clinical themes [18]. Only Aga Khan University Medical College in Pakistan offered a critical reading of research to third-year students in the late 1990s [19]. Another study at the University of Newcastle Medical School in 2001 suggested teaching critical evaluation to third-year students for only three weeks utilizing a JC and letter-writing strategy [8]. Yoon et al. recently evaluated how well EBM curricula were utilized in physical medicine and rehabilitation over the course of a year using the Assessing Competency in Evidence-Based Medicine (ACE) tool. Because of the increased workload and time required to comprehend such concerns, there was a drop in self-reported behavior and dissatisfaction. Only a few studies using EBM curricula have been reported in the literature, and these were largely for PGs and residents [5,6,20]. Furthermore, no consistent assessment approach or instrument was used in any of the aforementioned studies. The Fresno test and the Berlin questionnaire are standardized methods for measuring knowledge and abilities across three of the five EBM steps (ask, acquire, and appraise). The ACE tool, on the other hand, covers one to four steps of the EBM process. Observation is the best way to examine the fifth step [21].

A key barrier to obtaining such skills in the Core EPAs for graduating students published by the Association of American Medical Colleges is a lack of training in pre-clinical years and time constraints in clinical years for critical appraisal [6,15,20]. So far, no study has been undertaken among UG pre-clinical-year students, specifically the first year, to assess critical appraisal using a validated and reliable method. We hypothesize that including JCs in the first year of dental school aids in the early development of critical appraisal skills in students. To address this issue, we assigned JCs to dental students during their first year of study to aid in the development of critical thinking abilities. We then used the integrated ACE instrument to assess their performance and verified it in Pakistan, a developing Asian nation. This intervention in the curriculum is based on social learning, communities of practice, and distributive learning [22].

## Materials and methods

Study design and sampling technique

The present study was designed as a quasi-experimental study with non-probability convenience sampling.

Study setting

The study was conducted at the Dental College, HITEC Institute of Medical Sciences, Taxila, Pakistan. EBM was incorporated for pre-clinical students as a hybrid model in their curriculum with a blended learning approach. The curriculum spanned over a period of one year in 2021 for first-year dental students.

Ethical considerations

Ethical approval was sought and granted by the Ethical Review Board of HITEC Institute of Medical Sciences for this study (approval number: ERC/19/07). Informed consent was obtained from all participants at the start of the study.

Data collection

ACE Instrument

To assess the instrument’s adequacy concerning length and logic, a pilot research with a small sample of 15 participants was conducted. At the beginning of the study, students were given a PowerPoint presentation with challenging terminology and concepts taught to them to assist them in better understanding the questionnaire. The face validity and content of the ACE tool were determined through an iterative procedure by consensus expert opinion [23]. To evaluate the reliability and validity of the questionnaire, a test-retest methodology was used. Two weeks later, 15 students were invited to complete the same questionnaire once again to determine if their responses were similar.

A brief patient scenario and a clinical question were presented to users of the ACE tool. The next step was to give the users a search strategy and a sample article extract. Users needed to complete 15 questions by selecting yes or no, with each question corresponding to one of the four phases of EBM. Items 1 through 11 evaluated EBM-related knowledge and abilities, while items 12 through 15 evaluated EBM-related attitudes toward its application in clinical practice (Appendices). For the ACE tool, successful answers received a score of one, while erroneous answers received a score of zero, for a possible maximum of 15.

Intervention

Our study group included first-year dental students starting in 2021 (50 students) of similar demographic backgrounds, i.e., from Punjab, Pakistan. Currently, the hybrid system of teaching is in practice. The class of 50 students was divided into five groups of 10 students in each block. The student cohort was exposed to seven JCs in three blocks in a year. Each block comprised 10-11 weeks including the assessment. The JCs implemented in this hybrid model were relevant to the content delivered in physiology for each block for enhancing their understanding and integrating knowledge and skills acquired. As students were in their early-stage careers, they were provided with research articles for discussion in JCs.

The articles provided were on the following topics: Stokes-Adam syndrome, calcium signaling in myocytes, dental hemophilia, vitamin B12 deficiency, myasthenia gravis, tabes dorsalis, and tetanus. Students were provided with clear guidelines regarding the aims and objectives of the JCs including their structure and format by the faculty utilizing a PowerPoint presentation. The whole class was provided with the same article for the JCs. The groups were made for ease of discussion, participation, and presentation. JCs were conducted on the last Friday of each month. Sufficient time was provided for preparation for which students were notified at least a month before. Students prepared themselves in self-directed learning sessions. The timetable was adjusted in advance keeping in mind their academic preferences. On the day, 20 minutes were given for PowerPoint presentations followed by an interactive discussion with the class specifically focusing on the PICOTT (patient, intervention, comparison, outcome, type of question, and type of study). Each session also covered search tactics based on medical subject heading (MeSH) phrases and PubMed tutorials. At the end of each discussion, feedback was provided by the faculty based on 1-2 levels in Kirkpatrick’s training evaluation model, that is, reaction and learning [24].

After obtaining informed consent, the ACE tool was provided to the students at the start in January 2021 (pre-test) and end of the study/academic year in December 2021 (post-test). Each participant only filled out the questionnaire twice as a result. Students were required to work through 15 questions by answering yes or no [21]. Students answered the questionnaire under the direct supervision of one of the two authors. The study participants were assured that all responses would be anonymous and would only be used for improving the quality of future sessions and for dissemination in our professional community.

Data analysis

Data were analyzed using SPSS version 26 (IBM Corp., Armonk, NY). Inferential statistics were used for the comparative analysis of the ACE tool, namely, pre-test and post-test. The mean values with standard deviations of both the pre-test and post-test were obtained. Paired sample t-test was used to compare the mean test score (i.e., pre-test and post-test), where significance was assumed to be p < 0.05.

The difficulty index for each item was determined by adding all correct responses divided by the total number of students. Internal reliability was determined by corrected item-total correlation (CITC) using an average inter-item correlation test. A CITC ≥0.15 was considered acceptable as it examines the degree to which all test questions on the test measure a single construct [25]. Internal consistency was measured via Cronbach’s alpha. A Cronbach’s alpha ranging from 0.6 to 0.7 was considered to demonstrate acceptable internal consistency, 0.7-0.9 as good internal consistency, and >0.90 as excellent internal consistency. Item discrimination index (IDI) was determined and ranged from negative (-1) to positive (1), with a value of >0.2 considered adequate [22,26].

## Results

**Figure 1:**
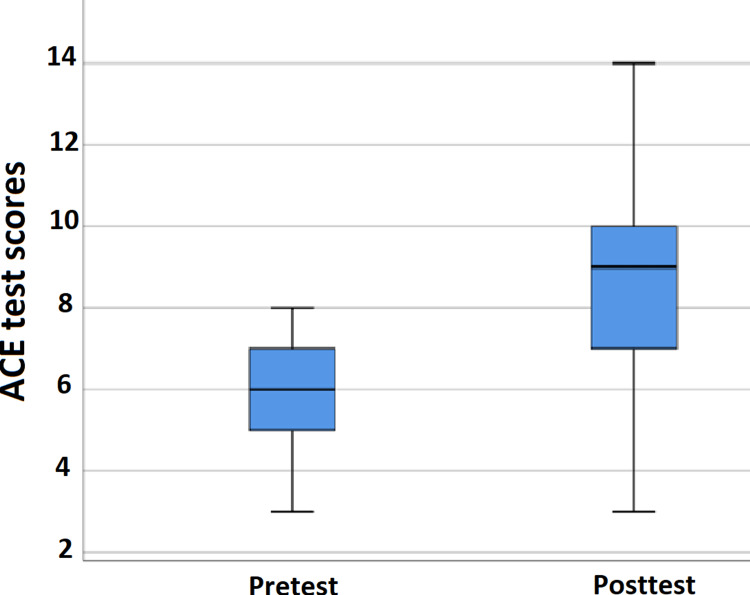
Box and whisker plot showing a linear trend of the ACE score (p < 0.001) for both pre-test and post-test with a median score of 6 and 9, respectively. ACE: Assessing Competency in Evidence-Based Medicine

The student cohort belonged to a similar age group with a mean age of 19 ± 1 years (range = 18-20 years) and a similar demographic background (i.e., from Punjab). In total, 43 (86%) of the respondents were females and seven (14%) were males. Using a box and whisker plot, a linear trend of median score from 6 in the pre-test to 9 in the post-test was observed (p < 0.001) (Figure 1). The post-test box plot shows data with a slight skewness toward the negative. The mean difference (95% confidence interval (CI) of the difference) between the pre-test and the post-test responses was -3.14 (-2.32 to -3.96) (p < 0.001) using a paired-sample t-test.

Regarding the post-test responses shown in Table 1, the difficulty index of each item was reasonably broad ranging from 0.3 to 0.9. For items six and eight, the item difficulty was 0.38 and 0.3, respectively. Internal reliability (CITC) was in the acceptable range (>0.15) for most of the items (range = 0.5-0.18). The CITC varied from 0.05 to 0.14 for items four, five, nine, 10, and 13 but all were positive. The IDI ranged from 0.8 to >0.2 for 12 items (1-3, 6-9, and 11-15) of the ACE tool. For the remaining three, the IDI was 0.2. None of the items showed a negative discrimination index. Regarding internal consistency that shows reliability, the Cronbach’s alpha was 0.64. This was considered acceptable internal consistency of items on the ACE tool.

**Table 1:** Pre-test and post-test individual item analysis showing item discrimination index, corrected item-total correlation, difficulty index, and p-value. The comparative values before and after the implementation of JCs show improvement in measured parameters with a significance of p = 0.001. Items 1-2 pertain to EBM step one (asking the answerable question), items 3-4 to step two (literature search), items 5-11 to step three (critical assessment), and items 12-15 to step four (applying the evidence to the patient scenario). ACE: Assessing Competency in Evidence-Based Medicine; EBM: evidence-based medicine; JC: journal club

Items (ACE tool)	Item discriminatory index		Corrected item-total correlation		Difficulty index (%)	
	Pre	Post	Pre	Post	Pre	Post
1	0.5	0.5	0.07	0.33	0.58	0.64
2	0.1	0.6	-0.40	0.30	0.48	0.62
3	0.3	0.6	-0.17	0.33	0.44	0.56
4	-0.1	0.2	-0.34	0.08	0.34	0.9
5	0.1	0.2	-0.32	0.11	0.3	0.86
6	-0.2	0.5	-0.47	0.25	0.42	0.38
7	0.2	0.5	-0.03	0.28	0.2	0.48
8	-0.1	0.6	-0.49	0.50	0.38	0.3
9	0.6	0.4	0.15	0.13	0.24	0.7
10	0	0.2	-0.23	0.14	0.28	0.4
11	0.8	0.7	0.23	0.36	0.46	0.56
12	-0.7	0.8	-0.66	0.41	0.4	0.48
13	0.8	0.3	0.25	0.05	0.34	0.72
14	0.1	0.6	-0.26	0.35	0.3	0.68
15	0.6	0.4	0.10	0.18	0.52	0.56

## Discussion

We report the effectiveness of JCs implemented in a hybrid model of the curriculum with a blended learning technique in dentistry for pre-clinical, first-year students using the ACE tool. We have implemented and tested the efficacy of the ACE tool in Pakistan where EBM is still taught differently in medical and dental institutions, especially in the early years of pre-clinical training. We have demonstrated a marked increase in the effectiveness of knowledge and skills acquired across the first four steps of the EBM process as per The Create Framework [27] in these students (Table 1, Figure 1) as the primary objective of this study.

Our study further shows that the ACE tool has acceptable validity and reliability as an instrument in assessing EBM competency in dental UG trainees (Cronbach’s alpha = 0.64). It offers a dichotomous outcome measure for assessing the first four steps of the EBM process. The pre-test and post-test IDI for the first two categories of the ACE tool (ask and acquire) for all constructs were able to discriminate well between the ones who were knowledgeable and who were not. In the appraisal category of the ACE tool having seven constructs, the items (6-8 and 10) showed a marked improvement in the discrimination index from -0.2 to 0.6. For the last category of applying the evidence, a couple of items (12 and 14) showed an improvement in the discrimination index from -0.7 to 0.8. When comparing pre-test and post-test results in Table 1, this quantitative assessment reveals improved comprehension of constructs in the ACE instrument. Similarly, a significant gain in EBM skills was found in three groups (novice - two years of training, intermediate - three years of training, and advanced - four years of training), including a spirally run EBM-based curriculum at Monash University, as measured by the ACE assessment [21]. Because no baseline measurements (pre-test) were taken in the Monash study, commenting on the extent of progress indicated is difficult. The difference in IDI values obtained for pre and post-test in our study is shown in Table 1. Another study involving the installation of JCs at PG training levels in physical medicine and rehabilitation found that the ACE tool helped to improve EBM proficiency. However, no statistically significant difference between the PG levels and average scores was found [28].

**Figure 2:**
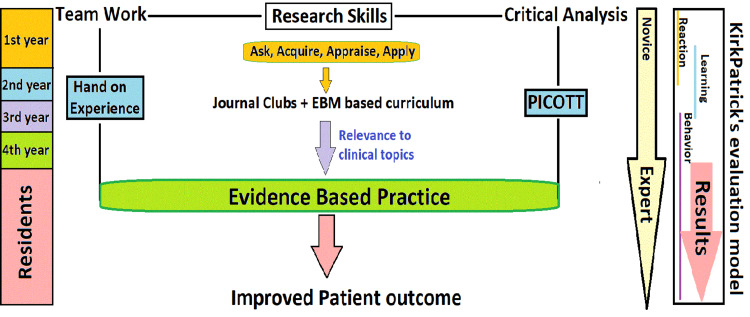
A conceptual map showing research skills development. Incorporation of JCs and EBM-based curriculum in pre-clinical years through the PICOTT and hands-on experience are used to instill scholastic, critical thinking, communication, and team working skills, which the World Health Organization defines as the five-star doctor’s general important skills. It shows evaluation at each stage based on Kirkpatrick’s model. JCs: journal clubs; EBM: evidence-based medicine; PICOTT: patient, intervention, comparison, outcome, type of question, and type of study

The CITC was good for more than 66% of the items tested with few exceptions. A low-point biserial (<0.15) means that students who got the item wrong also obtained a high overall score on the test, whereas students who got the item right had a low overall score [22,26]. In our testing, five of the items (4, 5, 9, 10, and 13) were found to be low performers in terms of internal reliability. More than 50% of these poor performers are related to critical appraisal. We believe that the research component of the first-year UG dentistry program, notably research methodology, biostatistics, and addressing the components of EBM, is lacking. The majority of EBM research has centered on either senior students (>two years) or PG education. Surprisingly, following a year of implementing the EBM curricula in PG training, a recent study found a decrease in self-reported behavior. Residents did express their dissatisfaction with the increased workload and time required to comprehend such topics [28]. Previous studies have reported similar results [5-7,9,13,21]. According to the authors, JCs and EBM-based curricula should be included in MBBS/BDS entry classes and run spirally. This pre-clinical curriculum revision would fill in the gaps in the first-year curriculum and provide additional reinforcement in clinical years through spiral integration, as shown in Figure 2.

Our results show that the items assessing the four levels of EBM on the ACE tool were of optimal level of difficulty ranging from 0.3 to 0.9. Such a difficulty index can discriminate the performance of poor and good students. This finding may be due to the broad sampling of knowledge content. Similar results were obtained while using the ACE tool in recent studies through the implementation of JCs in allied health and physical medicine and rehabilitation curriculum of postgraduates [28,29].

Based on the results of our study we propose a research skills development conceptual map from novices to residents for improved patient care. The basics on how to search for the best evidence are lacking both at UG and PG levels [30]. This map suggests that the acquisition of research skills program should be started from the first year of dental school and expanded in later years. The focus should be on an EBM-based curriculum with the implementation of JCs, problem-based learning, debates or role plays, and the formation of reading clubs. This will inculcate scholastic skills through PICOTT and hands-on experience with applied teaching and learning modalities. The main focus is to address and invoke critical thinking, team working, communication, professionalism, and risk management, as evidenced by CanMEDS 2015 physician competency framework, the Accreditation Council for Graduate Medical Education (ACGME), and the Association of American Medical Colleges’ Core EPAs [6,15,22]. The map also explains the evaluation at each step based on Kirkpatrick’s model [24].

Our study adopting Kirkpatrick’s evaluation methodology stands at the second level, i.e., learning through pre and post-learning assessment. We did not look for any evidence of behavior change as a result of participation in the JCs. Future research may be planned to look for behavioral transformation as part of the EBM process, as illustrated by Kirkpatrick’s model [24].

Limitations

This research was restricted to a single educational setting, which reduced the generalizability of the results. Therefore, any attempt to apply this conclusion to other educational settings must stay within the scope of the study. In addition to this, variables such as students’ prior academic achievement and psychological health were not controlled during the research, which could have affected the findings of the study.

## Conclusions

The use of JCs to increase active learning, critical appraisal, analytical, and decision-making skills was highly received by pre-clinical dental students. For testing EBM competency of dental students in Pakistan, the 15-item ACE tool is a relevant and reliable tool. Further integration of JCs into an EBM-based spiral curriculum from pre-clinical to clinical years will result in the desired outcomes, as described by CanMEDS, ACGME, and the World Health Organization’s Seven-Star Doctor’s generally important competencies.

## Appendices

**Table 2:** Assessing Competency in Evidence-Based Medicine (ACE) tool.

Asking an answerable question	Yes	No
1. Are all PICO elements described in the patient scenario?		
2. Does the question constructed post-scenario provide a focused, foreground question?		
Searching the literature	Yes	No
3. Will the search strategy (to be used in Medline) retrieve relevant studies relating to the question?		
4. Does the search strategy utilize appropriate MeSH/keywords and Boolean operators correctly and effectively?		
Appraising the evidence	Yes	No
5. Was there sufficient information to determine the representativeness of the study participants?		
6. Was the method of participant allocation to intervention/exposure and comparison adequate?		
7. Was any form of adjustment required?		
8. Were all participants blinded to the treatment/exposure?		
9. Were all investigators blinded to the treatment/exposure?		
10. Were all outcome assessors blinded to the treatment/exposure?		
11. Were all patients analyzed in the groups to which they were randomized?		
Applying the evidence	Yes	No
12. Does the patient in the scenario share similar characteristics/circumstances to participants in the study?		
13. Is the treatment/therapy feasible in the clinical setting of the scenario?		
14. Were all clinically important outcomes considered?		
15. Do the likely benefits of the treatment/therapy outweigh any potential harms and costs?		
